# Ammonia-Oxidizing Archaea and Bacteria Differentially Contribute to Ammonia Oxidation in Sediments from Adjacent Waters of Rushan Bay, China

**DOI:** 10.3389/fmicb.2018.00116

**Published:** 2018-02-02

**Authors:** Hui He, Yu Zhen, Tiezhu Mi, Lulu Fu, Zhigang Yu

**Affiliations:** ^1^College of Marine Life Science, Ocean University of China, Qingdao, China; ^2^Laboratory for Marine Ecology and Environmental Science, Qingdao National Laboratory for Marine Science and Technology, Qingdao, China; ^3^Key Laboratory of Marine Environment and Ecology, Ministry of Education, Qingdao, China; ^4^College of Environmental Science and Engineering, Ocean University of China, Qingdao, China; ^5^Key Laboratory of Marine Chemistry Theory and Technology, Ministry of Education, Qingdao, China

**Keywords:** pyrosequencing, ammonia-oxidizing archaea, ammonia-oxidizing bacteria, potential nitrification rates, sediment, Rushan Bay

## Abstract

Ammonia oxidation plays a significant role in the nitrogen cycle in marine sediments. Ammonia-oxidizing archaea (AOA) and bacteria (AOB) are the key contributors to ammonia oxidation, and their relative contribution to this process is one of the most important issues related to the nitrogen cycle in the ocean. In this study, the differential contributions of AOA and AOB to ammonia oxidation in surface sediments from adjacent waters of Rushan Bay were studied based on the ammonia monooxygenase (*amoA*) gene. Molecular biology techniques were used to analyze ammonia oxidizers’ community characteristics, and potential nitrification incubation was applied to understand the ammonia oxidizers’ community activity. The objective was to determine the community structure and activity of AOA and AOB in surface sediments from adjacent waters of Rushan Bay and to discuss the different contributions of AOA and AOB to ammonia oxidation during summer and winter seasons in the studied area. Pyrosequencing analysis revealed that the diversity of AOA was higher than that of AOB. The majority of AOA and AOB clustered into *Nitrosopumilus* and *Nitrosospira*, respectively, indicating that the *Nitrosopumilus* group and *Nitrosospira* groups may be more adaptable in studied sediments. The AOA community was closely correlated to temperature, salinity and ammonium concentration, whereas the AOB community showed a stronger correlation with temperature, chlorophyll-*a* content (chla) and nitrite concentration. qPCR results showed that both the abundance and the transcript abundance of AOA was consistently greater than that of AOB. AOA and AOB differentially contributed to ammonia oxidation in different seasons. AOB occupied the dominant position in mediating ammonia oxidation during summer, while AOA might play a dominant role in ammonia oxidation during winter.

## Introduction

Nitrification is one of the most essential steps in the nitrogen cycle, including the microbial oxidation of ammonia to nitrite and subsequently to nitrate. As the rate-limiting step in nitrification, ammonia oxidation is catalyzed by a series of phylogenetic and physiological microorganisms (Winogradsky, 1890; Venter et al., 2004; Könneke et al., 2005; Treusch et al., 2005). Before ammonia-oxidizing archaea (AOA) were found in Crenarchaeota in 2004, ammonia-oxidizing bacteria (AOB) were always considered the only contributors to ammonia oxidation. As a membrane-bound enzyme, ammonia monooxygenase (AMO) is an important functional protein for ammonia oxidation and can oxide ammonium in AOA and AOB (Rotthauwe et al., 1997; Könneke et al., 2005). By encoding the active site of ammonia monooxygenase, amoA can oxide ammonia and generate energy for the sequential oxidation reaction (Hooper et al., 1997; Junier et al., 2010). Thus, the *amoA* gene has been widely applied in molecular ecology research of AOA and AOB in different environments, such as lakes (Jiang et al., 2009; Hou et al., 2013b), wetlands (Ye et al., 2011; Sims et al., 2012a,b), soils (He et al., 2007; de Gannes et al., 2012; Wang et al., 2014; Zhao et al., 2015) and estuaries (Caffrey et al., 2007; Jin et al., 2011; Zheng et al., 2013). Compared with AOB, AOA predominates in many environments, indicating that the ammonia oxidation process promoted by AOA may be more intense (Shen et al., 2008; Jin et al., 2011; Hou et al., 2013a). The relative contribution of AOA and AOB to ammonia oxidation is one of the most important issues related to the nitrogen cycle in the ocean.

Studies have shown that salinity (Caffrey et al., 2007; Mosier and Francis, 2008; Santoro et al., 2008), ammonium concentration (Di et al., 2009; Niu et al., 2013; de Gannes et al., 2014) and dissolved oxygen concentration (Park et al., 2006; Santoro et al., 2008; Liu et al., 2011) are potential factors influencing the community structure of AOA and AOB. As electron acceptor and donor, oxygen and ammonia are essential for ammonia oxidation, but for AOA and AOB, the affinities of ammonia and oxygen differ. It has been found that AOA prefer areas with low ammonium concentrations while AOB prefer areas with high ammonium concentrations (Di et al., 2009), and several researchers have found that AOA show better survival than AOB under conditions of low dissolved oxygen (Kayee et al., 2011; Limpiyakorn et al., 2013). In addition to these factors, ammonia oxidizers’ communities were also affected by distinct seasonal variations. A previous study in Catalina Harbor showed that the AOA and AOB *amoA* gene copy numbers were greater in summer than in winter (Beman et al., 2012). In a seasonal study of AOA in the deep oligotrophic Lake Lucerne, the *amoA* gene copy numbers matched the seasonal dynamics within 16 months (Vissers et al., 2013). Seasonal changes in the *amoA* gene also reflected the dynamic changes in AOA community characteristics in oligotrophic high mountain lakes (Auguet et al., 2011).

Potential nitrification rates (PNR) are measured by the addition of excess ammonium under optimal conditions, and they can be applied to compare the different contributions of AOA and AOB to ammonia oxidation. Several studies have shown a correlation between PNR and *amoA* gene copy numbers. For example, PNR was positively related to the abundance of archaeal *amoA* gene, but it showed no significant correlation with bacterial *amoA* gene abundance in several southeastern regions of America (Caffrey et al., 2007). Wankel et al. (2011) were the first group to relate the *amoA* gene abundance to ^15^N isotope methods and found no obvious correlations between them in the Elkhorn Slough estuary. A positive correlation was also found between PNR and the archaeal *amoA* gene abundance in Yangtze estuarine sediments, suggesting that AOA might be more important for mediating ammonia oxidation in this area (Zheng et al., 2014).

Rushan Bay is a typical semi-enclosed sea and an important mariculture area in Shandong Peninsula, China. With the development of industry and aquaculture, large quantities of nitrogen (N) and phosphorous (P) nutrients have been transported into this area, contributing to a series of ecological problems such as eutrophication. Increasing nutrients and organic materials in estuarine and coastal waters can also result in an oxygen minimum zone (OMZ) or even hypoxia in the bottom water. An OMZ was previously found in Rushan Bay and its adjacent waters in 2009 (Cui et al., 2012; Ran et al., 2012). The presence of OMZ may destroy the oxidizing environment in surface sediment, which can impact the biogeochemical cycle of elements in marine environments, particularly for nitrification (Caffrey et al., 2007; Santoro et al., 2008; Qiu et al., 2013).

Sediments are habitats and can constitute enabling environments for microbes to flourish (Besaury et al., 2014). However, to date, there has been relatively little focus on the community structure and activity of AOA and AOB in surface sediments from adjacent waters of Rushan Bay. Moreover, a comprehensive understanding of the different contributions of AOA and AOB to ammonia oxidation in the studied sediments needs to be explored. In the present study, the composition, abundance and activity of AOA and AOB communities in sediments from adjacent waters of Rushan Bay were studied based on *amoA* gene sequences and potential nitrification incubation. Our main objectives were (1) to study the community structure and activity of AOA and AOB in surface sediments from adjacent waters of Rushan Bay, (2) to determine potential links between environmental factors and community structure, (3) to discuss the differential contributions of AOA and AOB to ammonia oxidation during summer and winter seasons in our studied area. We hypothesized that the (1) *Nitrosopumilus* group of AOA and *Nitrosospira* group of AOB would be more adaptable, exhibiting a marine influence to ammonia oxidizers’ communities, (2) AOA and AOB would contribute differentially to ammonia oxidation in different seasons owing to their different nitrification potentials and transcript abundance in different seasons.

## Materials and Methods

### Sediment Sampling

**FIGURE 1:**
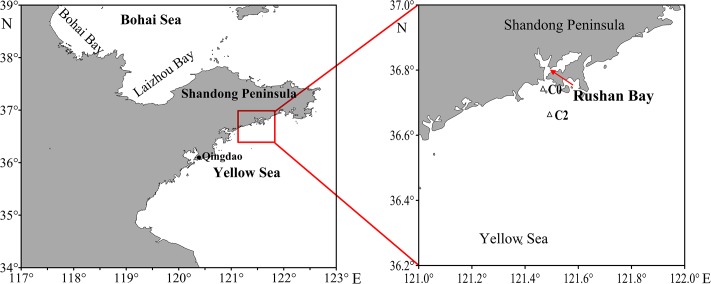
Location of sampling stations in adjacent waters of Rushan Bay, China.

The surface sediments and bottom water were collected from two stations (C0: 121.47°E, 36.74°N; C2: 121.49°E, 36.66°N) from adjacent waters of Rushan Bay during July (summer) 2014 and February (winter) 2015 (**Figure 1**). At each station, surface sediments (top 3 cm) were obtained using the box corer. After collection, all sediment samples were thawed, mixed thoroughly, and divided into two parts: one part was immediately stored at 4°C until potential nitrification incubation, and the other part was transported to the laboratory at -20°C and stored at -80°C for molecular analysis. The bottom water were filtered through 0.45-μm pore size polyethersulfone filters with N_2_ purging, and then immediately stored at -20°C for chemical analysis. Samples were labeled as summer (S) or winter (W).

### Environmental Factor Analysis

The physicochemical characteristics of the bottom water, such as salinity, temperature, chlorophyll-*a* content (chla) and dissolved oxygen concentration, were measured *in situ* with RBR XR-620 Multi-Channel CTD (Elcee). The remaining environmental factors (dissolved nitrogen and phosphate concentrations) were surveyed in the laboratory with a QuAAtro nutrient auto analyzer (Seal Analytical Ltd.).

### Potential Nitrification Rates

Potential nitrification rates were measured in triplicate according to the procedure of Bernhard et al. (2007) and Zheng et al. (2014). For each sample, a total of 1.0 g sediment was placed into 100-mL Erlenmeyer flasks with 30 mL seawater filtered using a 0.22-μm filtration membrane. All samples were amended with 300 μM ammonium (as NH_4_Cl) and 60 μM phosphate (as KH_2_PO_4_). The flasks were loosely covered, and then the suspension was incubated at 24°C (for summer) or 3.5°C (for winter), a temperature approximating that of the bottom water, with continuous shaking in the dark. During the incubation, subsamples were collected at 0, 24, 48, and 72 h of incubation, and then centrifuged and filtered for ammonium, nitrite and nitrate analysis. NH_4_^+^, NO_2_^-^ and NO_3_^-^ concentrations were measured using a QuAAtro nutrient auto analyzer. PNRs were calculated based on the changes in nitrate and nitrite concentrations over time.

To separate the archaeal and bacterial ammonia oxidation rates, an additional 1.0 g⋅L^-1^ ampicillin was placed in the flasks in a parallel experiment (Zheng et al., 2014). The experiment was conducted with three replicates per sample. As a beta-lactam antibiotic, ampicillin targets bacterial cell wall production when cells grow and thus can inhibit bacteria without affecting archaea (De Souza et al., 1999).

### Nucleic Acid Isolation and cDNA Synthesis by Reverse Transcription

DNA and RNA were extracted from each sediment sample using the PowerSoil^®^ DNA Isolation Kit (MoBio) and PowerSoil^®^ Total RNA Isolation Kit (MoBio), respectively. cDNA was synthesized with a Transcriptor First Strand cDNA Synthesis Kit (Roche). The extracted DNA and cDNA were stored at -20°C for further analysis. The DNA was used for pyrosequencing and qPCR, and the cDNA was used for qPCR.

### Quantitative PCR

Each DNA and cDNA extract was used for qPCR with the following primers: CamoA-19F (5′-ATG GTC TGG YTW AGA CG) and CamoA-616R (5′-GCC ATC CAB CKR TAN GTC CA) (Pester et al., 2012) for the AOA *amoA* genes and amoA-1F (5′-GGG GTT TCT ACT GGT GGT) and amoA-2R (5′-CCC CTC KGS AAA GCC TTC TTC) (Rotthauwe et al., 1997) for the AOB *amoA* genes. All qPCR assays were performed in triplicate with an ABI PRISM^®^ 7500 Sequence Detection System (Applied Biosystems) using the SYBR Green I method. The 20-μL qPCR reaction system contained 10 μL FastStart Universal SYBR Green Master (ROX) (Roche), 0.3 μM each primer, 0.2 μg⋅μL^-1^ bovine serum albumin (BSA) and 2.0 μL sediment DNA or cDNA. The qPCR amplification was started with an initial activation at 95°C for 10 min, followed by 40 cycles of 15 s at 95°C, 45 s at 54°C and 60 s at 72°C for AOA, or 40 cycles of 15 s at 95°C and 2 min at 58°C for AOB. After the amplification cycles, a melting stage was added to obtain a melting curve. Standard curves were generated using standard plasmids of AOA or AOB containing *amoA* genes.

The abundance and the transcript abundance of the *amoA* gene was examined using the above-mentioned qPCR protocols. In addition to the template DNA or cDNA, each reaction included serially diluted plasmids containing the target gene and negative control to ensure that the qPCR assay was stable and uncontaminated.

### Pyrosequencing

The community characteristics of AOA and AOB were studied using 454 pyrosequencing based on the *amoA* gene. The archaeal and bacterial *amoA* gene fragments were amplified using the specific primer pairs CamoA-19F/CamoA-616R and amoA-1F/amoA-2R, respectively. Barcodes were ligated to the 5′ ends of the primers to distinguish each sample. The reactions were held at 98°C for 5 min to denature the DNA, followed by 30 cycles of 98°C for 30 s, 50°C for 45 s and 72°C for 60 s and a final step at 72°C for 7 min. After purification with a SanPrep Column PCR Product Purification Kit (Sangon Biotechnology), pyrosequencing was conducted with the GS FLX+ Titanium platform at Personal Biotechnology in Shanghai, China.

The raw sequences obtained by pyrosequencing that exactly matched the barcodes and primers were retained. The sequences were subsequently trimmed to remove the barcodes and primers. Archaeal *amoA* sequences shorter than 425 nt and bacterial *amoA* sequences shorted than 450 nt were also excluded. After quality control, Uparse (Edgar, 2013) was employed to cluster the high-quality sequences into operational taxonomic units (OTUs) with a 97% similarity cutoff (Wang et al., 2015), and the most common sequence from each OTU were selected as the representative sequence.

### Statistical Analysis

The richness indices (Chao 1 estimator), diversity indices (Shannon index) and Good’s coverage were calculated using Quantitative Insights into Microbial Ecology (QIIME) (version 1.9.0). UPGMA clustering was conducted with the R program. Phylogenetic analysis was performed using MEGA6 (Tamura et al., 2013). Redundancy analysis (RDA) was employed to explore the correlations between microbial communities and environmental factors with CANOCO for Windows (version 4.5) (ter Braak and Šmilauer, 2002). Pearson’s correlation analysis for the abundance, transcript abundance, PNR and environmental factors were performed with SPSS statistics software (version 19.0), and the comparison of PNR among sampling stations and seasons was also archived using SPSS by Bonferroni correction.

### Accession Numbers of DNA Sequences

The raw data generated from pyrosequencing were deposited in the NCBI Sequence Read Archive (SRA) database under accession numbers SRP073125 and SRP073126.

## Results

### Environmental Factors at Sampling Stations

The environmental factors in the bottom water were shown in **Table 1**. The temperature was approximately 21.79–24.71°C in summer and decreased to 3.34–3.45°C in winter. An OMZ (3.7–7.0 mg⋅L^-1^) was observed in summer at our sampling stations. Salinity, chla and phosphate concentration were higher in winter than in summer. Ammonium and nitrate concentrations were both much higher than those of nitrite in both summer and winter.

**Table 1:** The environmental factors at the sampling stations.

Sample	Depth (m)	T (°C)	Sal	DO (mg⋅L^-1^)	Chla (μg⋅L^-1^)	NH_4_^+^ (μM)	NO_2_^-^ (μM)	NO_3_^-^ (μM)	PO_4_^3-^ (μM)
SC0	8.9	24.71	30.79	6.00	0.10	0.56	0.26	1.74	0.11
SC2	16.6	21.79	30.85	5.51	1.59	2.14	0.16	1.22	0.11
WC0	6.0	3.34	31.36	11.54	2.22	6.93	0.12	5.23	0.58
WC2	13.0	3.45	31.26	10.93	3.40	5.87	0.30	4.43	0.44

*T, temperature; *Sal*, salinity; *DO*, dissolved oxygen concentration; *Chla*, chlorophyll-*a* content*.

### *amoA* Gene Diversity

The Good’s coverage, Chao 1 estimator and Shannon index of AOA and AOB communities are listed in **Table 2**. The Chao 1 estimator and Shannon index indicated that AOA displayed higher richness and diversity than AOB at all sampling stations. Individually, AOA had a higher richness and diversity in summer, while AOB had a higher richness and diversity in winter. For each sample, AOA had the highest diversity at SC0, whereas AOB had the highest diversity at SC2. The coverage at all samples stations exceeded 0.99, suggesting that the *amoA* gene sequences retrieved from these samples could represent the majority of AOA and AOB communities.

**Table 2:** Richness indices, diversity indices and Good’s coverage of the AOA and AOB communities.

	AOA				AOB			
Sample	OTUs	Chao1	Shannon	Coverage	OTUs	Chao1	Shannon	Coverage
SC0	46	46	4.11	0.99	17	17	2.09	0.99
SC2	41	43.7	3.73	0.99	16	17	2.60	1.00
WC0	38	36.8	3.68	0.99	21	21	2.39	0.99
WC2	45	45.9	3.67	0.99	20	20	2.44	0.99

### AOA and AOB Community Compositions

**FIGURE 2:**
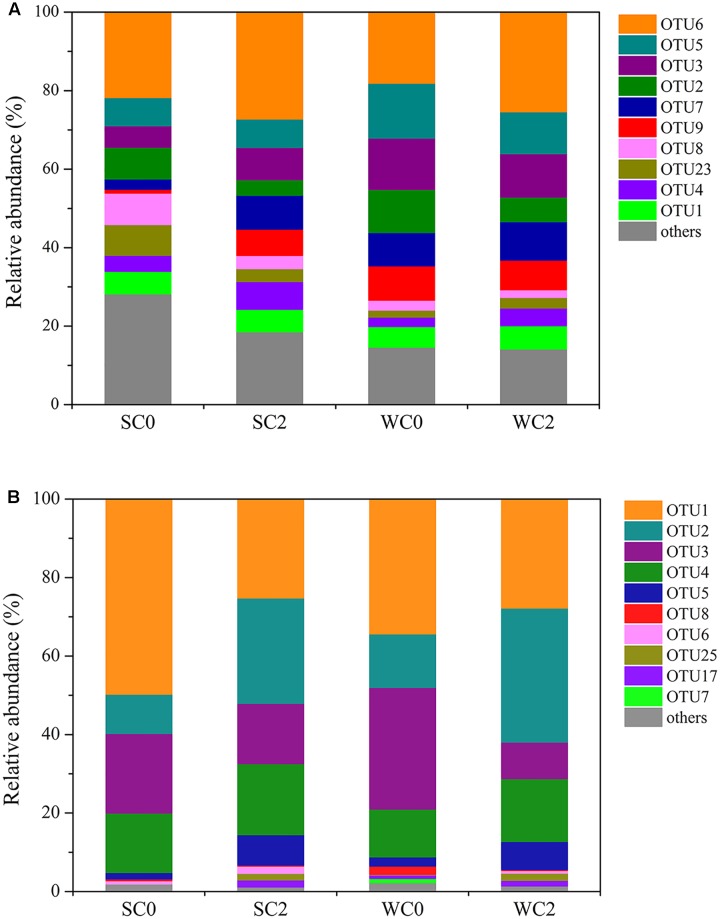
Relative abundance of AOA **(A)** and AOB **(B)** OTUs. Only OTUs with relative abundance rankings in the top 10 were analyzed.

Using a 3% cutoff, 59132 high-quality archaeal *amoA* gene sequences were clustered into 60 OTUs. Within each sample, the AOA OTU numbers ranged from 38 to 46, with the lowest richness at WC0 and the highest richness at SC0. The compositions of OTUs in the AOA communities are shown in **Figure 2A**, and only one predominant OTU (OTU6) was found. This result implies that AOA communities are generally ubiquitous and may be less influenced by environmental conditions.

**FIGURE 3:**
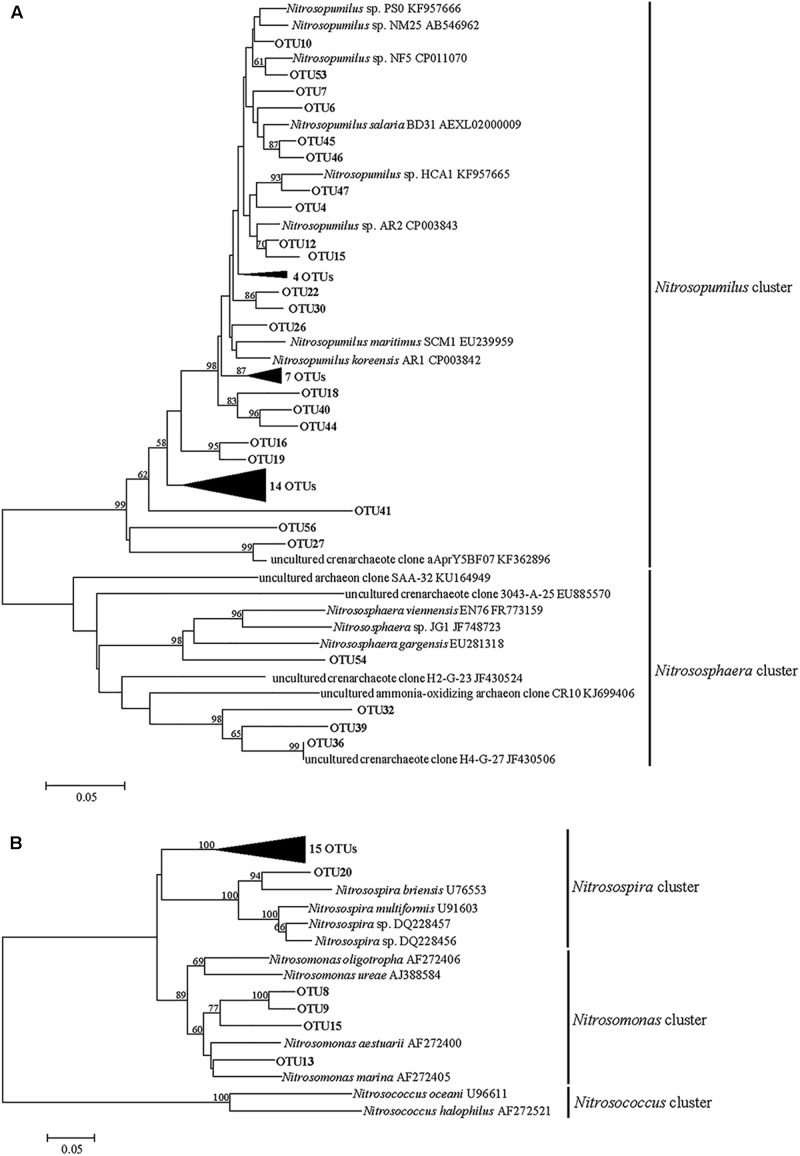
Neighbor-joining phylogenetic tree of archaeal **(A)** and bacterial **(B)** *amoA* gene sequences. Bootstrap values greater than 50% of 1000 resamplings are shown near nodes. The scale bar indicates 0.05 nucleotide substitutions per nucleotide position.

**FIGURE 4:**
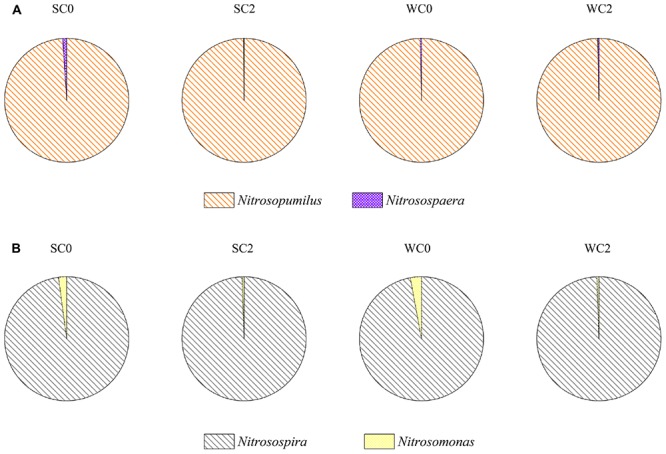
Distribution and relative abundance of phylogenetic AOA **(A)** and AOB **(B)** groups.

The neighbor-joining tree indicated that the AOA community was classified into two major groups in this study: *Nitrosopumilus* and *Nitrososphaera* (**Figure 3A**). Most of archaeal *amoA* gene sequences (more than 98.97% in each sample) fell within the *Nitrosopumilus* group. Only four OTUs were affiliated with the *Nitrososphaera* group, which was a subset of the soil/sediment cluster, and this group reached an abundance of 1.03, 0.13, 0.32, and 0.33% at SC0, SC2, WC0 and WC2, respectively (**Figure 4A**).

Regarding AOB, 43131 high-quality bacterial *amoA* gene sequences were grouped into 32 OTUs using the 3% cutoff. Within each sample, the AOB OTU numbers ranged from 16 to 21, with the lowest richness at SC2 and the highest richness at WC0. We discovered several predominant OTUs (**Figure 2B**), and the predominant OTU at each station was identical in summer and winter, with OTU1 at C0 and OTU2 at C2. Therefore, the dominant AOB species might change with environmental conditions, consistent with a previous study indicating that AOB could be used as an indicator of specific environmental conditions (Olsson et al., 2006; He et al., 2007).

Phylogenetic analysis showed that the AOB community was divided into the *Nitrosospira* group and *Nitrosomonas* group (**Figure 3B**). More than 97.11% of the bacterial *amoA* gene sequences fell within the *Nitrosospira* group, suggesting a significant role of *Nitrosospira* in the studied sediments. Compared with the *Nitrosospira* group, the *Nitrosomonas* group reached an abundance of 2.09, 0.48, 2.89, and 0.49% at SC0, SC2, WC0 and WC2, respectively, and the proportion of the *Nitrosomonas* group was greater at station C0 than at station C2 in both summer and winter (**Figure 4B**).

**FIGURE 5:**
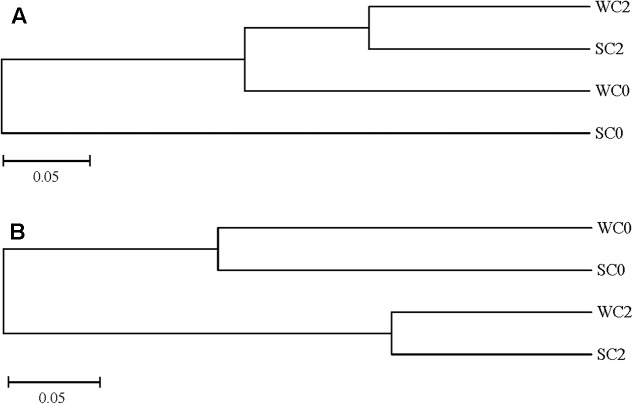
UPGMA clustering of AOA **(A)** and AOB **(B)** community compositions.

The variations in AOA and AOB communities were analyzed using UPGMA clustering. SC0 formed a single branch, and the other stations formed a branch, indicating that not only geographic locations but also seasons play a significant role in AOA community compositions (**Figure 5A**). For the AOB community, the UPGMA cluster tree showed that the AOB communities at station C0 were separately clustered from those at station C2 (**Figure 5B**), and it can be speculated that compared with the differences in seasons, different geographic locations had a greater effect on AOB community compositions. Our results also indicated that different seasons had a greater effect on the AOA community than on the AOB community.

### Impact of Environmental Factors on AOA and AOB Communities

**FIGURE 6:**
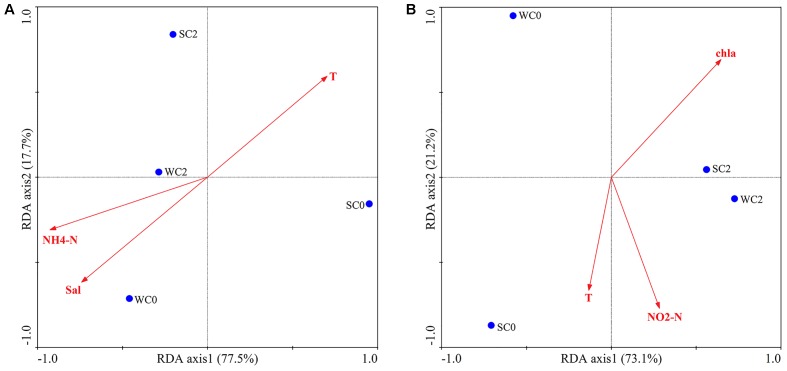
RDA ordination plots for the relationships between AOA **(A)** and AOB **(B)** communities and environmental factors.

The impact of environmental factors on AOA and AOB communities were determined via RDA. The OTUs of AOA and AOB communities were square root transformed and then used as species data for RDA analysis. The correlation was demonstrated by calculating the cosine value between the environmental factors and axes. BIOENV identified that temperature, salinity and ammonium concentration most strongly correlated with AOA communities, while temperature, chla and nitrite concentration most strongly correlated with AOB communities. The first two RDA dimensions explained 95.2% of the variation in the AOA community (**Figure 6A**). The first axis had the positive correlation with temperature (*r* = 0.7025) and negative correlation with salinity (*r* = -0.7405) and ammonium concentration (*r* = -0.9272). Thus, RDA analysis revealed that the AOA community was positively correlated with temperature and negatively with salinity and ammonium concentration. As for AOB, the first two axes represented 94.3% of the variation (**Figure 6B**). The first axis had the positive correlation with chla (*r* = 0.6922); for the second axis, it had the negative correlation with temperature (*r* = -0.6641) and nitrite concentration (*r* = -0.7701). Therefore, it was speculated that chla contributed greatly to the AOB community, followed by nitrite concentration and temperature. However, Monte Carlo permutations showed that none of the environmental factors significantly influenced AOA or AOB communities (*P* > 0.05, 499 Monte Carlo permutations).

### Abundance of *amoA* Genes

The reliability of the qPCR assay with both primers was confirmed by the strong linear inverse relationship between the threshold cycle value (C_t_) and the logarithmic value of the gene copy numbers (*R*^2^ > 0.99). Only one observable peak at the melting temperature (78.0°C for AOA and 79.3°C for AOB) was found. In addition, no primer-dimer artifacts or other non-specific PCR products were observed.

**FIGURE 7:**
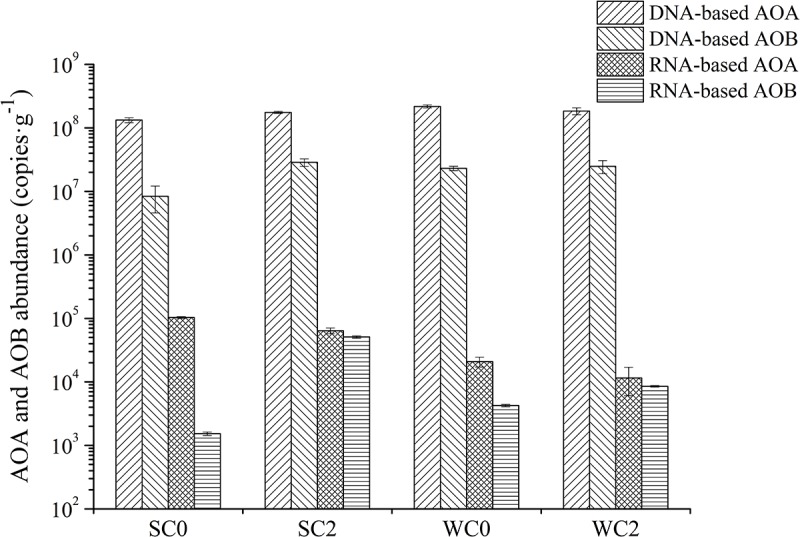
Quantitative analyses of AOA and AOB *amoA* gene abundance and transcript abundance. The error bars are the standard deviations of triplicate quantifications.

The abundance and the transcript abundance of archaeal and bacterial *amoA* genes was determined via qPCR (**Figure 7**). The amplification efficiencies were 81.69% for AOA and 99.54% for AOB. The abundance of archaeal *amoA* genes (ranging from 1.33 × 10^8^ to 2.18 × 10^8^ copies⋅g^-1^) was 1–2 orders of magnitude greater than that of bacterial *amoA* genes (ranging from 8.38 × 10^6^ to 4.80 × 10^7^ copies⋅g^-1^). Correlation analysis showed that no environmental factors appeared to contribute significantly to the abundance of archaeal or bacterial *amoA* genes (*P* > 0.05).

The transcript abundance of archaeal *amoA* gene (ranging from 1.15 × 10^4^ to 1.03 × 10^5^ copies⋅g^-1^) was also greater than that of bacterial *amoA* gene (ranging from 1.53 × 10^3^ to 2.63 × 10^4^ copies⋅g^-1^). The average transcript abundance of archaeal *amoA* was 8.37 × 10^4^ and 1.62 × 10^4^ copies⋅g^-1^ during summer and winter, respectively, and the average transcript abundance of bacterial *amoA* was also greater in summer than in winter, demonstrating that the ammonia oxidation process was more active during the summer. Based on the correlation analysis, temperature, chla and ammonium concentration were significantly related to the transcript abundance of archaeal *amoA* genes (*P* < 0.05).

### Potential Nitrification Rates

**FIGURE 8:**
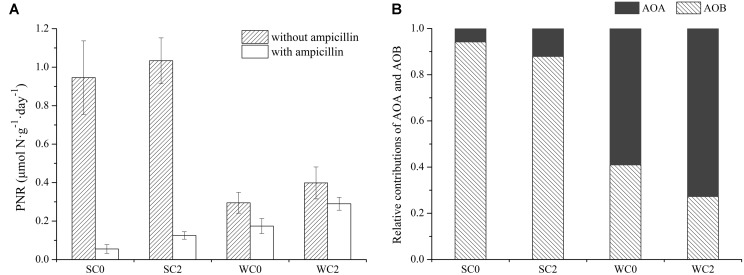
Potential nitrification rates (PNR) analyses in surface sediments from adjacent waters of Rushan Bay. **(A)** PNR in surface sediments from adjacent waters of Rushan Bay incubated with and without ampicillin. The error bars are the standard deviations of triplicate incubations. **(B)** The relative contributions of AOA and AOB to ammonia oxidation.

PNR in surface sediments from adjacent waters of Rushan Bay had significant seasonal variations (**Figure 8A**). Total PNR in summer was 0.95–1.03 μmol N⋅g^-1^⋅day^-1^, which was significantly greater than that in winter. The addition of ampicillin to separate the PNR of AOA from total PNR revealed that the PNR of AOA was much higher in winter, whereas the PNR of AOB was greater in summer. For total PNR and the PNR of AOB, a significant variation was observed among seasons (*P* < 0.05). However, sampling sites had no significant effect on total PNR, the PNR of AOA or the PNR of AOB (*P* > 0.05). Temperature, salinity, dissolved oxygen concentration, ammonium concentration, nitrate concentration and phosphate concentration had significant effects on the total PNR and PNR of AOB (*P* < 0.05), whereas a strong correlation was found between chla and PNR of AOA (*P* < 0.05). However, neither the *amoA* gene abundance nor its transcript abundance was significantly related to PNR in this study (*P* > 0.05).

Significant seasonal variations were detected in the relative contributions of AOA and AOB to ammonia oxidation (**Figure 8B**). The relative contribution of AOA in winter ranged from 59.07 to 72.79% and was significantly greater than that in summer. In contrast to AOA, the relative contribution of AOB was greater in summer, and the relative contribution of AOB in winter ranged only from 27.21 to 40.93%. Temperature, salinity and dissolved oxygen concentration had significant effects on the relative contribution of AOA and AOB to ammonia oxidation. Correlation analysis indicated that temperature was negatively related to the relative contribution of AOA (*P* < 0.05) but positively related to the relative contribution of AOB (*P* < 0.05). Salinity and dissolved oxygen concentration were significantly positively related to the relative contribution of AOA (*P* < 0.05) but negatively related to the relative contribution of AOB (*P* < 0.05).

## Discussion

Currently, most studies have used a cloning-sequencing method rather than a pyrosequencing method to examine AOA and AOB communities, and only a few studies have used pyrosequencing to describe *amoA* diversity (Pester et al., 2012; Mao et al., 2013; Hayashi et al., 2015; Tago et al., 2015; Zhang et al., 2016). In the present study, the community structure, diversity and activity of ammonia oxidizers were researched to understand the community information, the different contributions of AOA and AOB to ammonia oxidation and the impact of environmental factors on community composition in surface sediments from adjacent waters of Rushan Bay. AOB had a lower diversity than AOA, consistent with results from other coastal environments (Santoro et al., 2008; Cao et al., 2011b; Jin et al., 2011). Studies have identified that the diversity of AOA in estuaries might be greater than that in the adjacent open oceans, which may be due to nutrients in the river discharge (Llyd and Jody, 2003; Cao et al., 2011a). In the present study, the diversity of AOA at C0 was relatively greater than that at C2, consistent with the above view. Environmental factors, such as temperature, salinity, nitrate concentration and total nitrogen, were considered as driving forces affecting the diversity of AOA or AOB communities (Bernhard et al., 2005; Cao et al., 2011a; Yu et al., 2016). However, according to the correlation analysis, no noticeable environmental factors could be identified in relation to the diversity of AOA or AOB communities in our studied sediments (*P* > 0.05).

Phylogenetic analysis indicated that the AOA community was clustered into the *Nitrosopumilus* group and *Nitrososphaera* group (**Figure 3A**). A clear separation of AOA communities between marine and terrestrial environments was observed in our study, in accordance with a study of eastern intertidal sediments in Chongming Island (Zheng et al., 2013). The presence of soil-related AOA in our studied sediments could be explained by the deposition of microorganisms from upstream water with runoff, consistent with the greater relative abundance of *Nitrososphaera* group at C0 in both summer and winter (**Figure 4A**). The core microbiome of AOA communities exhibited greater similarity to the uncultured archaeal *amoA* gene sequences from other environments, such as the North Sea (Lipsewers et al., 2014), Tanoura Bay (Ando et al., 2009), Elkhorn Slough estuary (Wankel et al., 2011) and Eastern China Marginal Seas (Yu et al., 2016), showing that AOA obtained in this study was not representative of the special group in the studied sediments and their genotypes are ubiquitous worldwide.

The bacterial *amoA* sequences recovered from our studied areas were classified into the *Nitrosospira* group and *Nitrosomonas* group (**Figure 3B**). The *Nitrosospira* group accounted for most of AOB in our study, whereas the *Nitrosomonas* group showed lower relative abundance of 2.09, 0.48, 2.89, and 0.49% at SC0, SC2, WC0 and WC2, respectively (**Figure 4B**). The proportions of the *Nitrosomonas* group were greater at SC0 and WC0, suggesting that the upstream water has a significant impact on marine benthic microbes (Dang et al., 2008). Freitag et al. (2006) found that the *Nitrosospira* group dominated at stations with a marine influence, while the *Nitrosomonas* group had an advantage at stations with a freshwater influence. This evidence indicated that the *Nitrosospira* group may be more adaptable than the *Nitrosomonas* group in the coastal marine area, consistent with our results.

Various environmental factors can impact AOA and AOB communities, and their effects may be complicated. As the primary energy source of ammonia oxidizers, ammonium concentration is considered to be a vital factor influencing the ammonia oxidizers’ community structure. In a study of AOA communities in mangrove sediments, Li et al. (2010) demonstrated that ammonium concentrations might negatively impact the diversity of AOA. Ammonium concentrations were also found to be an important element for the community structure of AOB in intertidal sediments of Yangtze estuary (Zheng et al., 2014). Although AOB was sensitive to dissolved oxygen concentrations, studies have shown that dissolved oxygen concentrations can affect the activity of AOB rather than the AOB community (Park et al., 2008). Thus, no relationship between dissolved oxygen concentrations and the AOB community was found in the present study. RDA analysis indicated that temperature, salinity and ammonium concentration were the most important factors affecting the AOA community composition (**Figure 5A**), whereas temperature, chla and nitrite concentration appeared to play a pivotal role in the AOB community composition (**Figure 5B**). These factors have already been previously reported to be important for the determination of AOA and AOB communities (Sahan and Muyzer, 2008; Bouskill et al., 2012). Moreover, RDA indicated that no single environmental factor was likely to completely determine the AOA or AOB community compositions (*P* > 0.05, 499 Monte Carlo permutations), and the ammonia oxidizers’ community structure in the present study seemed to be influenced by several environmental factors in combination, possibly due to a variety of nature environmental gradients and multicollinearity among many abiotic factors in coastal estuarine regions.

**FIGURE 9:**
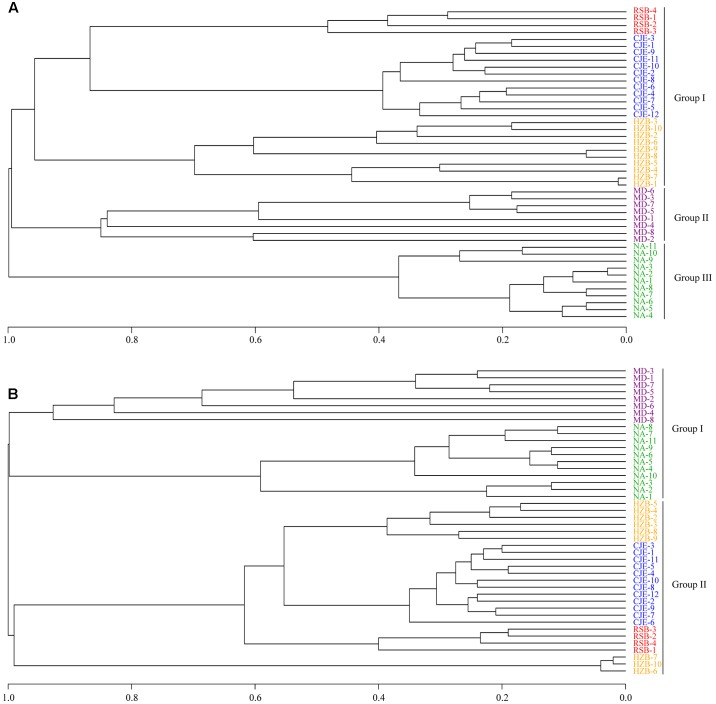
UPGMA clustering of AOA **(A)** and AOB **(B)** community compositions in previous studies and in the present study. RSB-^∗^, CJE-^∗^, HZB-^∗^, MD-^∗^, and NA-^∗^ indicate sequences from this study, Changjiang Estuary (Xia et al., 2015), Hangzhou Bay (Zhang et al., 2015, 2016), Minami-Daito Island (Hayashi et al., 2015) and Ny-Ålesund (Tago et al., 2015), respectively.

The *amoA* gene obtained in the present study and other five pyrosequencing studies (sediments from Changjiang Estuary and Hangzhou Bay, soils from Minami-Daito Island and Ny-Ålesund) (Hayashi et al., 2015; Tago et al., 2015; Xia et al., 2015; Zhang et al., 2015, 2016) were trimmed as reported above. The remaining high-quality sequences were clustered into 280 OTUs (for AOA) and 201 OTUs (for AOB) at 97% similarity levels. UPGMA clustering of the samples from the five environments was conducted using the R program. The UPGMA cluster showed a clear separation of AOA and AOB communities in samples from these five environments. The community types of AOA were clustered into three different groups consisting of group I, group II and group III (**Figure 9A**). The environment samples from Rushan Bay (RSB), Changjiang Estuary (CJE) and Hangzhou Bay (HZB) showed marine characteristics and were grouped together, while the other two soil samples formed group II and group III. Therefore, we speculated that geography contributes significantly to the community structure of AOA, consistent with reports showing that diverse and distinct AOA communities are associated with each habitat (Francis et al., 2005; Dang et al., 2009). Furthermore, similar to the UPGMA clustering of AOA communities, the AOB communities were clustered into two different groups according to the sediment and soil habitats (**Figure 9B**). The environment samples from RSB, CJE and HZB, which showed marine characteristics, were grouped together. The AOB community from soils in Minami-Daito Island (MD) appeared to be more similar to that in Ny-Ålesund (NA), although they are far from each other geographically. Many studies have shown that AOB communities are dominated by *Nitrosomonas* in terrestrial environments, whereas in estuarine areas, *Nitrosospira* may be more adaptable (Bartosch et al., 2002; Dang et al., 2010; Cao et al., 2011a). Based on all of these results, we can speculate that geography also contributes significantly to the community structure of AOB. The difference in geography mainly reflects differences in environmental conditions. Thus, the role of geography in the ammonia oxidizers’ community structure essentially depends on different environmental factors, for instance, ammonium concentration, salinity and temperature.

Consistent with most reports, our study showed that AOA was more abundant than AOB (Liu et al., 2011; Yao et al., 2011; Sims et al., 2012a,b; Hou et al., 2013b). Environmental factors, for instance, salinity, temperature and ammonium concentration, have been identified as potential factors to determine the abundance of AOA and AOB. In our study, correlation analysis indicated that no single environmental factor was likely to completely determine the ammonia oxidizers’ abundance, which seemed to be influenced by a combination of factors. The abundance of *amoA* gene could provide information concerning the potential ammonia-oxidizing activity, while the transcript activity of the *amoA* gene is a measure of protein production. In the studied sediments, both the ratios of active AOA to total AOA and the ratios of active AOB to total AOB were less than 1%, which indicated that most of the AOA and AOB *amoA* genes are likely associated with inactive cells. Studies have shown that external ammonium concentrations can up-regulate the expression of the *amo* operon. For example, in *Nitrosomonas europaea*, the proximal *amoCAB* promoter is active only when recovery from ammonium starvation, while the distal *amoCAB* promoter is constitutively active in the presence of ammonium (Berube et al., 2007). Our finding that the transcript abundance of the archaeal *amoA* gene peaked in the lowest ammonium concentration region was consistent with previous findings. To regulate the archaeal *amoA* gene transcription, temperature is another critical element (Tourna et al., 2008). Greater archaeal *amoA* transcript abundances were observed in warmer seawaters in our studied sediments, but no significant relationship was observed between archaeal *amoA* transcripts and temperature (*P* > 0.05).

Potential nitrification rates can reflect potential ammonia-oxidizing activity. There may be some certain connections between PNR and the abundance of ammonia oxidizers; however, the relationships did not achieve significant levels in our study (*P* > 0.05). Many environmental factors, such as salinity, ammonia concentration, dissolved oxygen concentration and temperature, can impact PNR. In the present study, there was a strong negative correlation between the total PNR and salinity, indicating that a high level of salinity could inhibit the metabolic activity of ammonia oxidizers. In addition, the total PNR also showed a strong positive correlation with temperature, which could explain why the total PNR were higher in summer. In addition to the total PNR, the PNR of AOB and temperature also showed a significant positive correlation (*P* < 0.01). All previously identified AOB are mesophilic bacteria, and their optimum growth temperature usually ranges from 20°C to 30°C (Yu et al., 2015). Temperature can directly affect the nitrification activity of AOB, which peaks at 15°C to 25°C (Avrahami et al., 2003). Enzyme activity decreases with decreasing temperature, ultimately resulting in a decline in the nitrification activity of AOB in winter. In contrast to AOB, AOA has a wide tolerance to temperature (Schleper et al., 2005; Jiang et al., 2010), and thus AOA shows an obvious competitive advantage over AOB at a high or low temperature.

Although the abundance and transcript abundance of AOA were greater than that of AOB in both summer and winter, the PNR of AOB and AOA was greater in summer and winter, respectively, leading us to hypothesize that PNR is directly related to nitrification potentials and the transcript abundance of ammonia oxidizers. Studies have shown that the nitrification potentials of AOB are 1–3 times as many as AOA (Jia and Conrad, 2009). Temperature was one of the important factors affecting nitrification potentials in this study (*P* < 0.05). The seasonal variation of the nitrification potentials directly led to the seasonal differences in the relative contributions of AOA and AOB to ammonia oxidation. The nitrification potentials of AOB decreased during winter, and AOB’s transcript abundance was relative lower. Thus, AOA dominated ammonia oxidation in surface sediments from adjacent waters of Rushan Bay. In summer, AOA had a greater transcript abundance than AOB, while AOB had far higher nitrification potentials than AOA. Thus, AOB contributed significantly to ammonia oxidation in surface sediments from adjacent waters of Rushan Bay.

## Conclusion

In general, in surface sediments from adjacent waters of Rushan Bay, the diversity of AOA was higher than that of AOB. *Nitrosopumilus* group of AOA and *Nitrosospira* group of AOB may be more adaptable in the studied sediments. Both the abundance and the transcript abundance of AOA were greater than those of AOB, and most AOA and AOB *amoA* genes are likely associated with inactive cells. Significant seasonal variations were observed in PNR, and AOA and AOB contributed differentially to ammonia oxidation in different seasons. AOB occupied the dominant position in mediating ammonia oxidation during summer, while AOA might be dominant in ammonia oxidation during winter.

## Author Contributions

HH, YZ, TM, and ZY designed the experiments. HH and LF performed the experiments and analyzed the data. HH and YZ wrote the paper.

## Conflict of Interest Statement

The authors declare that the research was conducted in the absence of any commercial or financial relationships that could be construed as a potential conflict of interest.
